# Heme oxygenase-1 in macrophages controls prostate cancer progression

**DOI:** 10.18632/oncotarget.5284

**Published:** 2015-09-16

**Authors:** Zsuzsanna Nemeth, Mailin Li, Eva Csizmadia, Balazs Döme, Martin Johansson, Jenny Liao Persson, Pankaj Seth, Leo Otterbein, Barbara Wegiel

**Affiliations:** ^1^ Department of Surgery, Beth Israel Deaconess Medical Center, Harvard Medical School, Boston, MA, USA; ^2^ Department of Medicine, Beth Israel Deaconess Medical Center, Harvard Medical School, Boston, MA, USA; ^3^ Cancer Center, Beth Israel Deaconess Medical Center, Harvard Medical School, Boston, MA, USA; ^4^ Transplant Institute, Beth Israel Deaconess Medical Center, Harvard Medical School, Boston, MA, USA; ^5^ Department of Laboratory Medicine, Lund University, Malmo, Sweden; ^6^ Department of Tumor Biology, National Koranyi Institute of TB and Pulmonology, Budapest, Hungary; ^7^ Division of Thoracic Surgery, Medical University of Vienna, Vienna, Austria; ^8^ Department of Thoracic Surgery, National Institute of Oncology, Budapest, Hungary

**Keywords:** tumor-associated macrophages, mitochondria, heme oxygenase-1, E-cadherin, tumor microenvironment

## Abstract

Innate immune cells strongly influence cancer growth and progression via multiple mechanisms including regulation of epithelial to mesenchymal transition (EMT). In this study, we investigated whether expression of the metabolic gene, heme oxygenase-1 (HO-1) in tumor microenvironment imparts significant effects on prostate cancer progression.

We showed that HO-1 is expressed in MARCO-positive macrophages in prostate cancer (PCa) xenografts and human prostate cancers. We demonstrated that macrophage specific (*LyzM-Cre*) conditional deletion of HO-1 suppressed growth of PC3 xenografts *in vivo* and delayed progression of prostate intraepithelial neoplasia (PIN) in TRAMP mice. However, initiation and progression of cancer xenografts in the presence of macrophages lacking HO-1 resulted in loss of E-cadherin, a known marker of poor prognosis as well as EMT. Application of CO, a product of HO-1 catalysis, increased levels of E-cadherin in the adherens junctions between cancer cells. We further showed that HO-1-driven expression of E-cadherin in cancer cells cultured in the presence of macrophages is dependent on mitochondrial activity of cancer cells.

In summary, these data suggest that HO-1-derived CO from tumor-associated macrophages influences, in part, E-cadherin expression and thus tumor initiation and progression.

## INTRODUCTION

The tumor microenvironment provides unique conditions to regulate cellular transformation and cancer growth [1, 2]. Understanding the balance of signals produced by stromal immune cells in the tumor microenvironment facilitates new targeted therapies for cancer treatment [3, 4]. Tumor-associated macrophages (TAMs) and infiltrating myeloid cells constitute the main components of the tumor stroma and are commonly designated as M2 polarized or alternatively activated macrophages (AAM) [1, 2, 5].

Epithelial to mesenchymal transition (EMT) is characterized by loss of E-cadherin and thus loss of cell adhesion, gain of migratory capacity, and increased invasiveness, stemness, and resistance to apoptosis [6, 7]. EMT generally occurs at the invasive front of metastatic tumors where TAMs accumulate [8] and has been linked to high expression of Twist and Snail [9], changes in mitochondrial activity, reactive oxygen species (ROS) production, and modulation of glucose and lipid metabolism [10, 11]. Knockdown of mitochondrial complex I increased ROS production and led to enhanced migration, invasion, and spheroid formation [12]. Similarly, a mutation in the NADH dehydrogenase subunit 6 generates a deficiency in respiratory complex I, leading to ROS overproduction and enhanced metastatic potential of cancer cells [13]. We recently showed that carbon monoxide (CO) requires functional mitochondria to induce cell cycle arrest and apoptosis of prostate cancer xenografts [14, 15]. However, the link between regulation of cancer metabolism and specifically how heme degradation pathways influence tumor-associated macrophages and EMT in cancer cells remains poorly defined.

Heme oxygenase-1 (HO-1) is a metabolic protein responsible for the degradation of heme to carbon monoxide (CO), bilirubin, and iron [16]. Although the role of HO-1 in macrophages in models of sepsis and injury is well-recognized, HO-1 expression in the tumor microenvironment remains to be investigated [17]. We have recently shown that HO-1 is a critical regulator of myeloid cell differentiation of CD14^high^ macrophages [18]. Others and we have shown that HO-1 is present in the nucleus of moderately differentiated tumors and is associated with tumor progression [19–21]. HO-1 induction favors epithelial phenotype by preventing the loss of E-cadherin and increased α-smooth muscle actin expression in rat renal fibrosis [22]. In line with this observation, treatment with heme fostered an E-cadherin/β-catenin interaction at cell-cell junctions and high induction of HO-1 by heme was associated with increased levels of E-cadherin [23]. Further, beneficial effects of HO-1 on reversing EMT were demonstrated in human peritoneal mesothelial cells [24].

In this study, we evaluated the role of HO-1 in myeloid cells in regulation of tumor growth and progression. Further, we hypothesized that HO-1 controls coupling of metabolism and respiration between cancer cells and macrophages. We demonstrated a role for HO-1 in immune cell in tumor progression via regulation of mitochondrial activity and expression of E-cadherin in tumor cells.

## RESULTS

### HO-1 is expressed in tumor-associated macrophages

**Figure 1:**
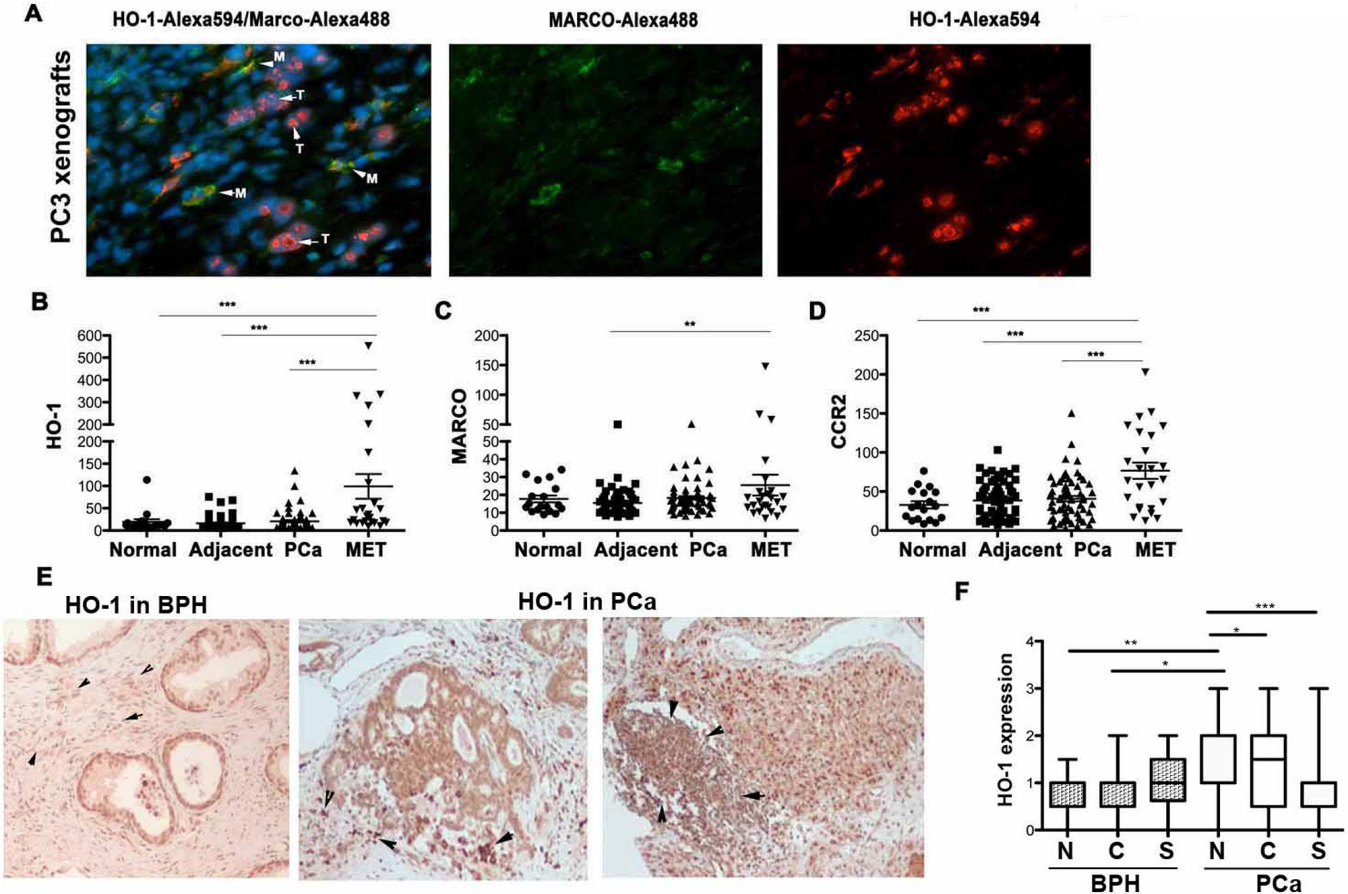
HO-1 is expressed in tumor microenvironment **A.** PC3 xenografts were established in nude mice for 4 weeks. Immunofluorescence staining with antibody against HO-1 and MARCO (macrophage marker) was performed. Arrows indicate cells co-stained with antibodies against HO-1 (Alexa549, Red) and MARCO (Alexa488, Green) and Hoechst (nuclei staining). M-macrophages, T-tumor cells. Note that cancer cells express nuclear HO-1 and TAM express cytoplasmic, enzymatically active HO-1. **B–D.** GEO mRNA expression profiles from 18 normal prostatic tissues, 62 tissues adjacent to tumors, 64 primary prostate tumors (PCa) and 24 prostate cancer metastases (MET) as described in [26] and Material and Methods. Mean values ± SD are shown. **p < 0.01, ***p < 0.001. **E–F.** Immunohistochemistry with antibody against HO-1 in prostate cancer and BPH (benign prostatic hyperplasia) biopsies. Representative stromal expression of HO-1 is shown in E and quantification is shown in F.

Others and we have previously demonstrated that HO-1 is highly expressed in prostate cancer [14, 15] and that HO-1 in cancer cells is targeted to the nucleus and remains enzymatically inactive to drive a malignant cell phenotype [25]. Overexpression of enzymatically active HO-1 in A549 lung carcinoma cells blocks tumor xenograft growth as compared to xenografts established from A549 cells stably transfected with control empty vector (control vector: 406 ± 58 mm^3^, HO-1: 134 ± 28.4 mm^3^, *p* < 0.05). However, because macrophages expressed high levels of HO-1, we reasoned that HO-1 in myeloid cells in the tumor microenvironment may also influence tumor growth and spread. We found that in addition to nuclear HO-1 in cancer cells in tumor xenografts, HO-1 is also expressed in stromal cells, specifically in the cytoplasm of MARCO positive macrophages in tumors (Figure 1A). HO-1 was not present in F4.80^high^ or MMR^high^ macrophages in tumor stroma (data not shown).

Since immune cells are important for the invasion and metastatic spread of tumors, we first correlated HO-1 expression with macrophage markers. We evaluated HO-1 mRNA expression in available GEO profiles from 18 normal prostate tissues, 62 samples adjacent to tumor tissue, 64 primary prostate tumors and 24 prostate cancer metastases [26]. We found significantly higher expression of HO-1 in metastatic prostate cancer samples as compared to normal tissues and primary tumor samples (Figure 1B). Similarly, we showed increased expression of TAM markers such as MARCO and CCR2 in metastatic biopsies as compared to primary cancer specimens (Figure 1C–1D). We demonstrated a significant and positive correlation between HO-1 and MARCO expressions in all samples (Table 1).

**Table 1:** Correlation between the mRNA expression of HO-1 and macrophage markers: CCR2 and MARCO in patients with prostate cancer

	MARCO	CCR2	HO-1
MARCO		0.1570051*	0.2149287**
CCR2	0.1570051*		0.1448182
HO-1	0.2149287**	0.1448182	

* *p* < 0.05

** *p* < 0.01

We have further evaluated HO-1 protein expression in human prostate cancer and BPH biopsies, which allowed us to gain information about HO-1 levels and location in tumor and stroma cells. We confirmed that HO-1 is expressed in both cancer and stromal cells as well as in infiltrating immune cells (Figure 1E–1F). Interestingly, we found that HO-1 expression in the nuclei of tumor cells was significantly higher in cancer versus benign prostatic hyperplasia (BPH), while lower expression of HO-1 was detected in stromal cells as compare to nuclear staining in PCa (Figure 1F). However, only few samples in our cohort showed infiltration of leukocytes and therefore we were unable to correlate the levels of HO-1 in immune cells with clinical parameters.

These data suggest that changes in the expression of HO-1 both in the nucleus as well as in stroma might influence cancer progression.

### TAM-derived HO-1 expressed in differentiated macrophages modulates tumor initiation and progression

**Figure 2:**
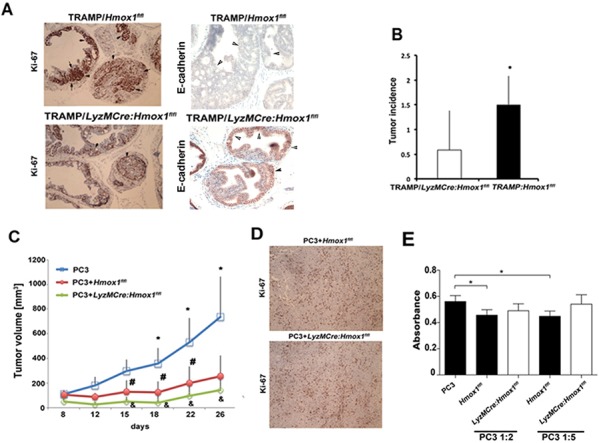
TAM-derived HO-1 modulates prostate cancer progression **A–B.** *Hmox-1^fl/fl^* mice were crossed with *LyzM-Cre* mice to generate myeloid linage specific knockout of HO-1 (*LyzM-Cre:Hmox1^fl/fl^*), which were further crossed to TRAMP mice. Tumor initiation was measured at 25 weeks of age by evaluation of Ki67 positivity within the prostate glands. Sections were also stained with E-cadherin, loss of which is a marker of tumor progression. Number of glands per field of view (FOV) with prostatic intraepithelial neoplasia (PIN) or adenocarcinoma lesions were evaluated as incidence of tumorigenesis based on H&E staining in *n* = 5–6 mice per group. Data ± SD are shown, *p < 0.05 (one-tailed *t*-test). **C–D.** PC3 cells or PC3 cells mixed with BMDM from *Hmox1^fl/fl^* and *LyzM-Cre:Hmox1^fl/fl^* mice were inoculated into the flanks of nude mice. Xenograft growth was measured over time (C) and Ki67 staining (D) was performed at day 26 after inoculation. *p < 0.01, PC3 versus PC3+ BMDM from *Hmox1fl/fl* mice and #*p* < 0.05, PC3+ BMDM from *Hmox1^fl/fl^* versus PC3+ BMDM from *LyzM-Cre:Hmox1^fl/fl^*; *n* = 3–5 mice per group. **E.** BrdU incorporation assay was employed to measure proliferation of PC3 co-cultured with BMDM from *LyzM-Cre:Hmox1^fl/fl^* and *Hmox^fl/fl^* mice in the ratio of 1:2 or 1:5 (PC3:BMDM) for 24 h. Data are representative for 3 independent experiments in triplicates. *p < 0.05.

To investigate the role of HO-1 in tumor stroma with focus on TAMs during tumor development and progression we have used the well-established model of prostate cancer in TRAMP mice [15], which were crossed to knockout mice with specific deletion of HO-1 in myeloid cells (Figure 2A–2B). Evaluation of prostate intraepithelial neoplasia (PIN) and cancer lesions in TRAMP mice at age 25 weeks demonstrated significant tumorigenesis when crossed to *Hmox1^flfl^* mice but much lower tumor incidence in TRAMP:*LyzM-Cre-Hmox1^flfl^* mice (Figure 2A–2B). Higher Ki67 staining indicative of cancer proliferation was detected in *Hmox1^flfl^* mice and correlated with low expression of E-cadherin, a marker of cancer progression (Figure 2A). Similarly, inoculation of mouse lung carcinoma cells (CRL), into the flanks of *LyzM-Cre:Hmox1^flfl^* showed slower growth of syngeneic tumor transplants as compared to CRL tumors in *Hmox1^flfl^* mice (% of tumor growth at day 12: *Hmox1^flfl^*: 489.5 ± 109.6, *LyzM-Cre:Hmox1^flfl^*: 300.6 ± 111.11, *p* = 0.06, *n* = 3 mice per group with 2–3 tumors per mouse), suggesting the importance of HO-1 in tumor microenvironment.

To directly test a role of differentiated macrophage-derived HO-1 in regulation of tumor development, we inoculated PC3 cells with bone marrow derived macrophages (BMDM) expressing HO-1 (PC3+*Hmox1^flfl^*) or lacking HO-1 (PC3+*LyzM-Cre:Hmox1^flfl^*) into the flanks of nude mice. Surprisingly, the presence of differentiated macrophages in PC3 xenografts resulted in significantly slower growth of tumors as compared to PC3 cells derived-xenografts (Figure 2C). Deletion of HO-1 in myeloid cells (*LyzM-Cre-Hmox1^flfl^*) further significantly suppressed tumor growth as shown by Ki67 staining and growth curve evaluation (Figure 2C–2D, #*p* < 0.05, PC3+*Hmox1^flfl^* versus PC3+*LyzM-Cre:Hmox1^flfl^*). These data together with the direct deletion of HO-1 in myeloid cells in TRAMP model suggest that HO-1 in macrophages is a positive regulator of tumor growth or transformation due to induction of M2 polarization [27–29], change in macrophage differentiation [18] or influence on other immune cells in tumor microenvironment.

*In vitro* co-culture of PC3 cell with macrophages expressing HO-1 (*Hmox1^flfl)^*) or lacking HO-1 (*LyzM-Cre-Hmox1^flfl^*) demonstrated that HO-1 expressing macrophages significantly suppressed tumor growth after 24 h compared to PC3 control, however macrophages lacking HO-1 failed to significantly alter growth of PC3 cells (Figure 2E). This suggests that presence or infiltration of other cell types in the tumor microenvironment may dictate the suppressive effect of macrophages lacking HO-1 *in vivo*.

### HO-1 in macrophages controls immune cell infiltration to the tumor microenvironment

**Figure 3:**
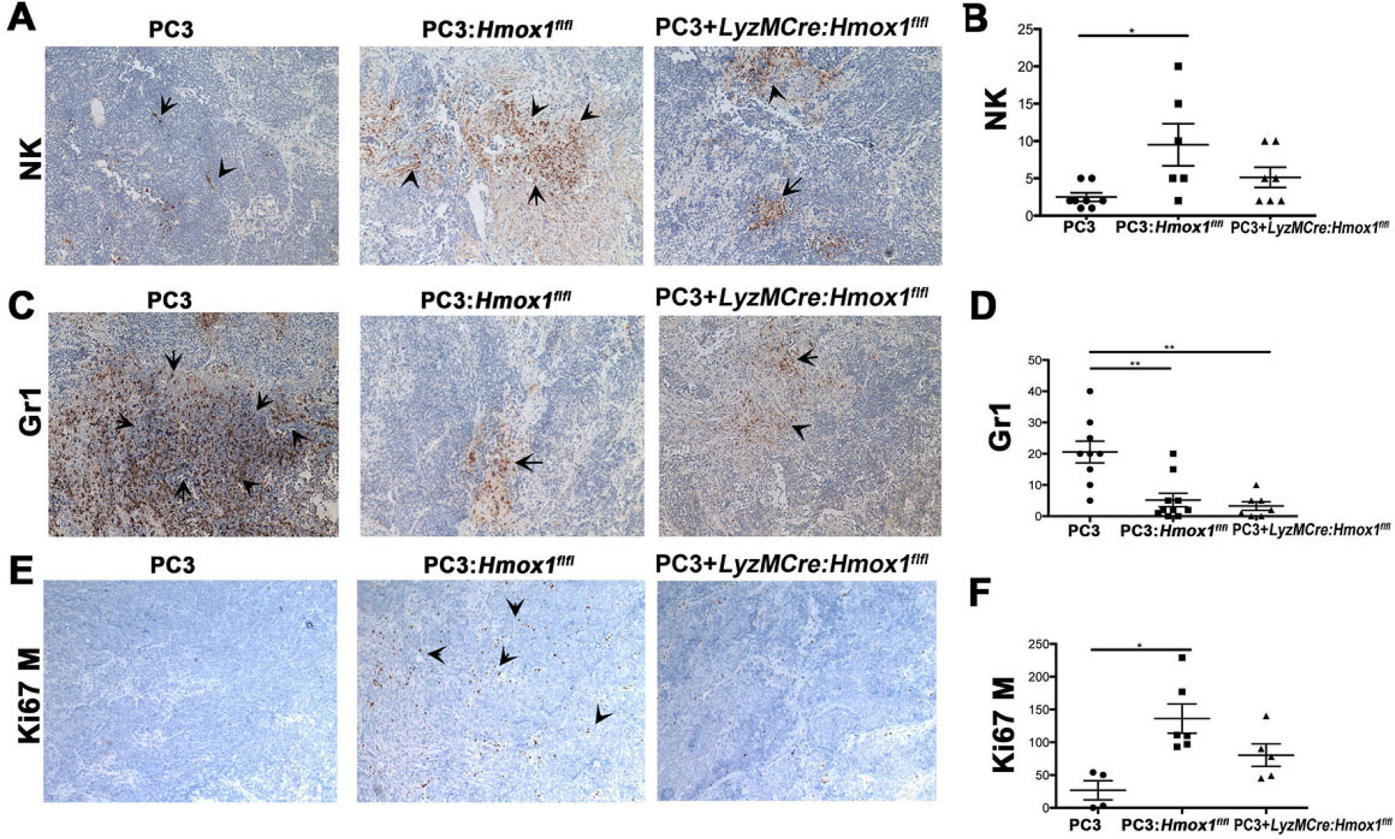
Presence of HO-1-postitive macrophages in the tumor microenvironment modulates infiltration of Gr1+ and NK cells **A–F.** Immunohistochemistry of xenografts established from PC3 cells alone or mixed with BMDM (1:1) from *Hmox1^fl/fl^* and *LyzM-Cre:Hmox1^fl/fl^* mice in nude mice as in Figure 2. NK cells (NKp46, A–B), granulocyte (Gr-1, C–D) or proliferation (mouse Ki67) (E–F) markers were evaluated by immunohistochemistry. Quantization of staining is expressed as number of positive cells (Ki67, Gr1) or % of area of staining (NKp46, Gr1) presented from *n* = 5–6 FOV (B, D, F). *n* = 3–4 animals per group. Data are presented as mean values ±SD.

Since cancer growth is controlled by multiple cell types in the tumor microenvironment, we tested whether HO-1 in macrophages could influence infiltration of immune cells and thus affect tumor development. Decreased growth of tumor xenografts in the presence of macrophages was associated with high infiltration of NK cells and decreased infiltration of Gr-1 positive granulocytic cells (CD11b negative) (Figure 3A–3D). The presence of macrophages in tumor xenografts corresponded to higher number of Ki67 positive infiltrating leukocytes into the tumor (Figure 3E–3F) suggesting expansion of infiltrating cells in the tumor. In contrast, in PC3 xenografts injected with myeloid cells with deletion of HO-1, the number of NK and Ki67 positive cells did not increase as compared to PC3 xenograft alone (Figure 3E–3F). However, there was a significant inhibition in number of Gr-1 positive cells in xenografts with the absence of HO-1 in macrophages suggesting the specificity of HO-1 in controlling NK cell infiltration (Figure 3).

### TAMs control EMT *in vivo*

**Figure 4:**
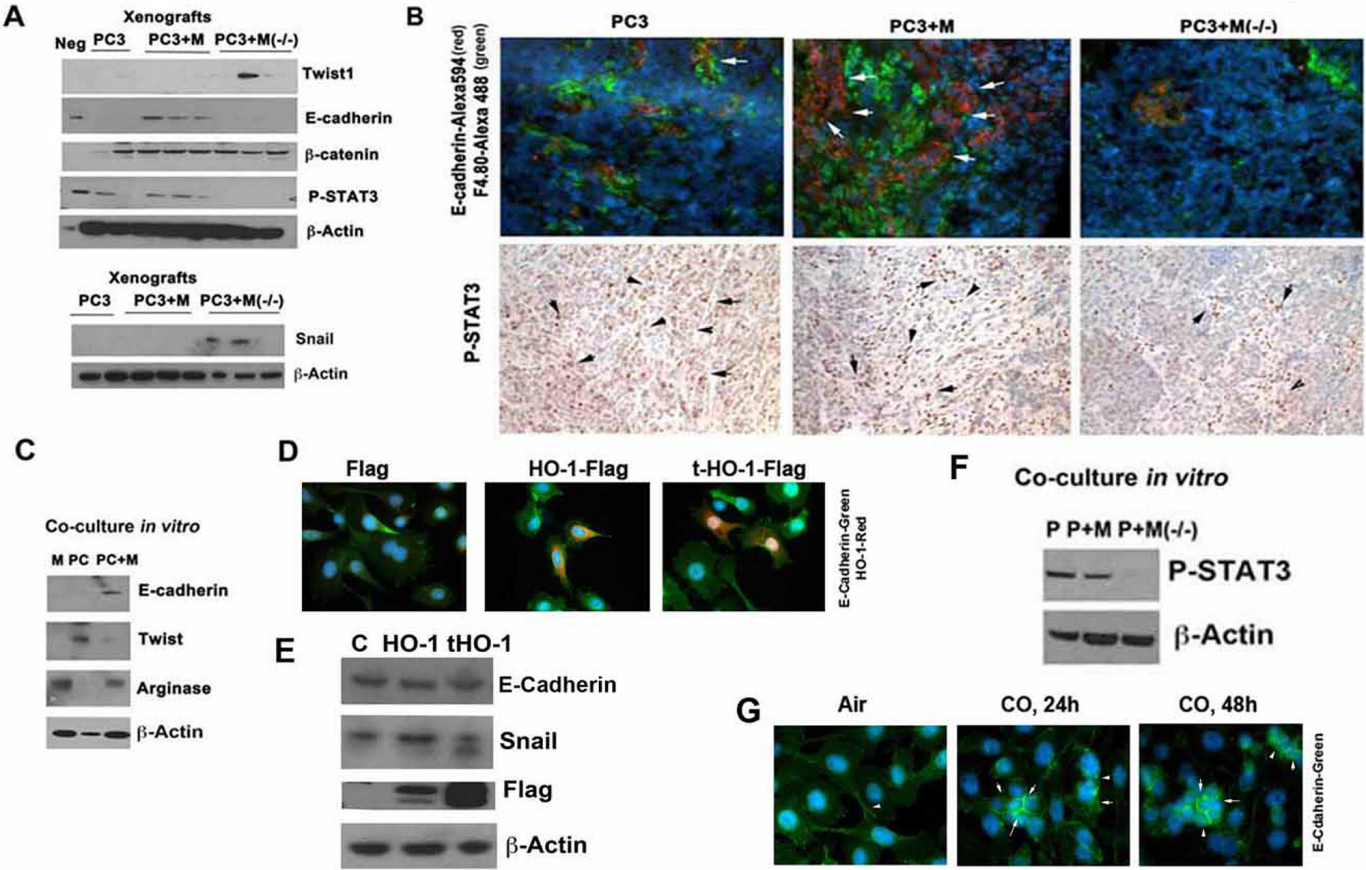
TAM-derived HO-1 reverted EMT in other words triggered mesenchymal to epithelial transition MET *in vivo* **A.** PC3 cells alone or PC3 mixed with BMDM from *Hmox1^fl/fl^* (M) and *LyzM-Cre:Hmox1^fl/fl^* (M−/−) mice were inoculated into the flanks of nude mice as described in Figure 3 and xenografts were harvested on day 26. Immunoblotting with antibody against twist1, E-cadherin, Snail and β-catenin was performed. Neg: normal skin. **B.** Immunostaining and immunohistochemistry with antibody against E-cadherin (Alexa 594-red) and F4.80, a marker of macrophages (Alexa488-green) were applied in the xenografts as above. **C.** PC3 (PC) cells were co-cultured with BMDM (M) and harvested after 24 h. Immunoblotting with antibody against E-cadherin, twist1 and arginase was performed in the lysates of the co-cultures. Immunoblots correspond to the mixture of proteins isolated from both cell types. **D–E.** PC-3 cells were transfected with HO-1 (cytoplasmic) and truncated HO-1 (cytoplasmic and nuclear) and the levels of E-cadherin and HO-1 were correlated by immunostaining (HO-1-Alexa549, E-cadherin-Alexa488) or immunoblotting. Data are representative for 3 independent experiments. **F.** PC3 cells co-cultured *in vitro* with BMDM from *Hmox1^fl/fl^* (M) and *LyzM-Cre:Hmox1^fl/fl^* (M−/−) mice were harvested after 24 h and lysates were immunoblotted for HO-1 (m-mouse, h-human) and P-STAT3. Data are representative for 3 independent experiments. **G.** PC3 cells were treated with exogenous CO (250 ppm) for 24 or 48 h and the levels of E-cadherin were measured by immunofluorescence staining. Data are representative for 3 independent experiments. Arrows indicate positive staining for E-cadherin.

Since the control of tumor growth by macrophages was unexpected, we next asked whether differentiated myeloid cells with high HO-1 expression influences EMT. Co-inoculation of macrophages with cancer cells allow for re-expression of E-cadherin, a marker of less aggressive epithelial cancer phenotype. Interestingly, lack of HO-1 in macrophages resulted in decreased expression of E-cadherin (Figure 4A) and increased mesenchymal markers Twist-1 and Snail as compared to macrophages with high HO-1 expression (Figure 4A–4C). In contrast, overexpression of full length or truncated (partially located in the nucleus) HO-1 in PC3 cells did not significantly altered expression of E-cadherin (Figure 4D–4E). This suggests that lack of HO-1 in myeloid cells rather than in cancer cells in the co-culture model of differentiated macrophages and cancer cells may slow tumor growth while leading to more aggressive and invasive cancer. Inverse correlation between cancer growth and invasive phenotype has been previously described [30, 31].

### HO-1 derived CO targets STAT3 and mitochondrial pathways to control EMT

Since STAT3 was shown to mediate the effects of HO-1-derived and exogenous CO [32], we next looked at the effects of HO-1 in tumor microenvironment on STAT3 signaling. We found that lack of HO-1 in myeloid cells resulted in lower activation of STAT3 in the co-culture of PC3 and BMDM *in vivo* and *in vitro* as measured by decreased levels of phosphorylated STAT3 (Figure 4A, 4B, 4D, 4F).

**Figure 5:**
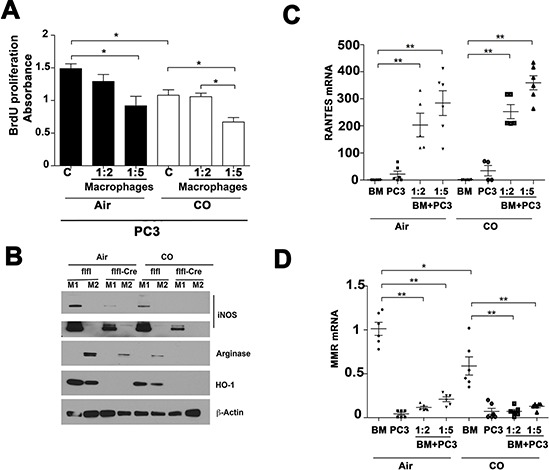
Effects of CO treatment and HO-1 in macrophages on PC3 cell growth **A.** BrdU incorporation assay was employed to measure proliferation of PC3 cocultured with bone marrow derived macrophages (BMDM) and treated with CO (250 ppm) for 24 h. *p < 0.05. Absorbance at 450 nm corresponds to the number of proliferating cells. **B.** *LyzM-Cre:Hmox1^fl/fl^* (flfl-Cre) and *Hmoxfl/fl* (flfl) BMDM were polarized for 3 days with IL-4 (M2 phenotype) or LPS/INFγ (M1 phenotype) in the presence or absence of CO treatment (250 ppm) and arginase (M2 marker), iNOS (M1 marker) and HO-1 were measured by immunoblotting. **C–D.** Real time PCR with primers against RANTES and MMR in BMDM (BM) from *LyzM-Cre:Hmox1^fl/fl^* and *Hmoxfl/fl* mice co-cultured with PC3 cells for 24 h in the presence or absence of CO. *p < 0.05, **p < 0.01.

The majority of the effects of HO-1 are mediated by the generation of one or more heme degradation products. Therefore, we also tested whether CO influenced macrophage-mediated effects on PC3 cells (Figure 4G & 5). Treatment with CO for 24–48 h resulted in higher expression of E-cadherin as compared to air treated PC3 cells (Figure 4G). Further, exposure of PC3 to CO at 250 ppm for 24 h resulted in suppression of cancer cell growth (Figure 5A). Similarly, BMDM expressing HO-1 blocked cancer cell growth, which was further inhibited by CO (Figure 5A–5C). We previously showed that CO suppresses growth of human xenografts as well as TRAMP and KRAS-driven tumors in mice [15].

We next tested whether HO-1-derived CO and exogenous CO modulates macrophage phenotype when co-cultured with cancer cells. Lack of HO-1 leads to lower arginase (M2 marker) and iNOS (M1 marker) expression as compared to wild type BMDM (Figure 5B) suggesting the role of HO-1 in maturation and polarization of macrophages [18]. Both markers were further suppressed by application of exogenous CO (Figure 5B). Evaluation of RANTES and mannose receptor (MMR) mRNA levels in macrophages co-cultured with PC3 cells showed higher levels of RANTES and lower mannose receptor (MMR), indicating M1 macrophage polarization in the presence of PC3 cancer cells, and that this polarization was in part augmented by CO (Figure 5C–5D). These data suggest that inefficient macrophage polarization in the absence of HO-1 or CO may be a contributing factor to the effects on cancer growth and EMT.

**Figure 6:**
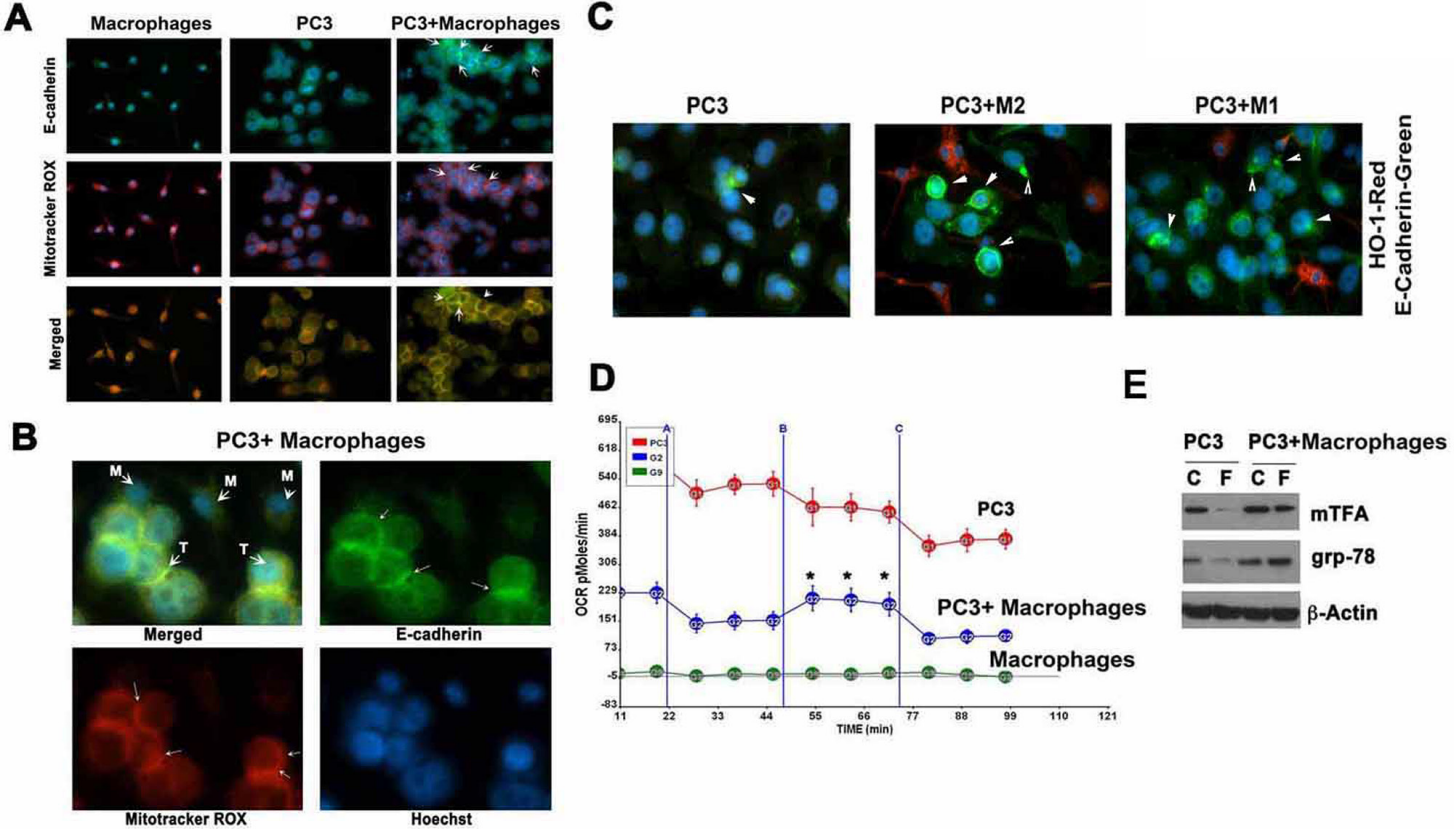
Macrophages regulate mitochondrial respiration of cancer cells **A–B.** Co-culture of PC3 and BMDM from *Hmox1^fl/fl^* for 24 h were stained using Mitotracker Red CMXRos for detection of mitochondria and with anti-E-cadherin antibody. Note that presence of BMDM in the culture increased E-cadherin expression in the membrane that corresponded to the higher intensity of staining with Mitotracker Red. **C.** Co-culture of PC3 cells with M1 or M2 BMDM in a 1:2 ratio. Immnofluoresence staining with antibody against HO-1 (Alexa 549, macrophage staining) and E-cadherin (Alexa488, PC3 cells staining) was performed. Data are representative for 2 independent experiments. Arrows indicate positive staining. **D.** Seahorse XF Cell Mito Stress Test was performed in cultures of PC3, PC3+macrophages and macrophages alone. Administration of oligomycin (vertical lane A), FCCP (vertical lane B) and rotenone/antimycinA (vertical lane C) was followed by real time measurements of OCR (oxygen consumption rate) by Seahorse. *p-0.01, PC3 versus PC3+macrophages. **E.** The levels of mtchTFA and grp-78 in the culture of PC3 or PC3+BMDM treated with FCCP (F; Mesoxalonitrile 4-trifluoromethoxyphenylhydrazone, 0.1 mM) for 24 h were evaluated by immunoblotting. Data are representative for 3 independent experiments in triplicates.

In efforts to understand how macrophages were influencing EMT, we tested the role of mitochondrial respiration, which is a target for HO-1-derived CO or exogenous CO. We used Mitotracker probe that labels active mitochondria (Figure 6A–6B) and showed that mitochondrial staining was much stronger under the cell membrane in PC3 cells co-cultured with BMDM as compared to PC3 alone (Figure 6A–6B). Further, presence of macrophages increased expression of E-cadherin on the cell surface. To evaluate whether differential polarization of macrophages influenced E-cadherin expression, we co-cultured M1-like or M2-like macrophages with PC3 cells. We showed that M2-like macrophages strongly induced E-cadherin expression on PC3 cells, while M1-like macrophages only slightly upregulated E-cadherin on the surface of cancer cells (Figure 6C).

We reasoned that there might be a correlation between higher mitochondria activity and E-cadherin assembly in PC3 cells in the presence of macrophages. We found that the presence of macrophages resulted in higher internal respiratory capacity in cancer cells in response to the mitochondrial stressor FCCP as compared to PC3 cells or macrophages cultured alone (Figure 6D). Indeed the presence of macrophages in the co-culture experiments resulted in restoration of expression of the mitochondrial transcription factor A (mTFA) and grp-78 that were otherwise suppressed after treatment with FCCP (Figure 6E).

### Negative correlation between HO-1 and E-cadherin in patient samples

**Figure 7:**
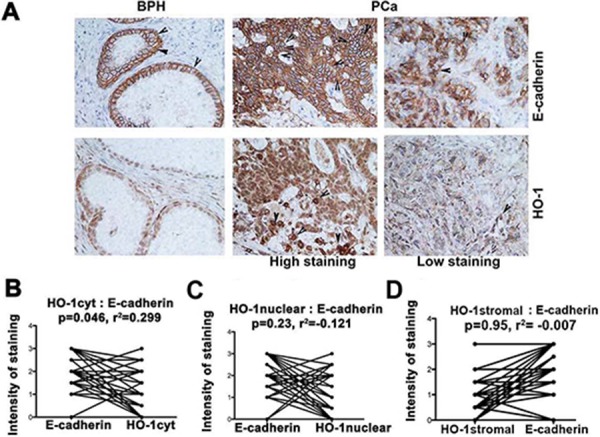
HO-1 expression correlates with E-cadherin levels in prostate cancer (PCa) and metastatic cancers **A–D.** Expressions of HO-1 and E-cadherin were evaluated in consecutive sections of 35 prostate cancer and corresponding BPH samples. Cytoplasmic, nuclear and stromal HO-1 were evaluated and correlated with E-cadherin expression in the samples. Note: Cytoplasmic but not nuclear or stromal HO-1 correlates with E-cadherin expression.

To evaluate the clinical significance of our studies on the role of HO-1 in regulation of EMT, we compared expression of HO-1 and E-cadherin in primary BPH and prostate tumors. We show a strong positive correlation between expression of cytoplasmic HO-1 and E-cadherin (Figure 7A–7B). There was no significant correlation between nuclear tumor-derived HO-1 or stromal HO-1 with expression of E-cadherin (Figure 7C–7D). However, lower stromal expression of HO-1 in tumor biopsies as compared to BPH may indicate metastatic switch, which leads to loss of E-cadherin in primary tumors (Figure 1). High nuclear expression of HO-1 in cancer cells may be an independent driver of invasion [33] while cytoplasmic HO-1 or myeloid cells-derived HO-1/CO may suppress EMT and cancer progression [15, 34].

## DISCUSSION

Immune cells in the tumor microenvironment shape multiple properties of cancer cells including their invasive phenotype, proliferation and survival. In this study, we asked whether and how the metabolic enzyme HO-1 influences the TAM phenotype and thus influences cancer cell growth and metastasis.

In this study, we identified HO-1 in myeloid cells to be an important regulator of tumor progression and EMT. We show that cell-specific deletion of HO-1 in myeloid cells suppressed tumor initiation and growth while increasing EMT in different models of prostate cancer. Furthermore, we identified mitochondrial respiration in macrophages as a modulator of EMT and E-cadherin expression.

We found that HO-1 was expressed in MARCO positive macrophages in established tumors and that there was a strong correlation between both HO-1 and MARCO expression in prostate cancer metastases. MARCO is a class I scavenger receptor that mediates antibody-dependent phagocytosis of pathogen as well as of cancer cells and is expressed in M2 macrophages [35]. Presence of HO-1 in MARCO-positive macrophages may indicate the mechanism for regulation of tumor growth via enhanced polarization of macrophages towards a pro-angiogenic M2 phenotype. Expression of HO-1 did not correlate with levels of CCR2, a macrophage receptor, which allows for monocyte recruitment into tumors and metastatic spread of breast cancer in mice models as well as marker of M1 polarization [36]. Indeed, cytoplasmic HO-1 correlated with higher level of E-cadherin, accordingly to the studies by Gueron et al. showing that heme induced E-cadherin and change in cell morphology in prostate cancer cells [23].

Polarization of macrophages towards an M1 or M2 phenotype dictates the immune response of T cells as well as angiogenesis and metastatic growth. We found that the presence of mature macrophages in the microenvironment at time of tumor initiation delays growth of spontaneous tumors, syngeneic transplants or xenografts. Number of differentiated macrophages in the tumor microenvironment might be a limiting factor that allows for dormancy of tumors and suppression of development of advanced and metastatic disease. Depletion of HO-1 from differentiated TAMs (in xenograft model) further suppressed the growth of cancer cells but initiated EMT. Indeed, HO-1 in TAMs has been associated with accelerated tumor growth in breast cancer [37]. HO-1 is a strong inhibitor of acute inflammation and therefore may block inflammation in cancer [38]. Further, HO-1 regulates myeloid differentiation [18] as well as immune response to viral [39] or bacterial infection via increased IL-1β release [40]. HO-1 in the tumor niche promoted lung metastases by controlling VEGF and IL-10 production [41]. The balance between proinflammatory signaling and anti-inflammatory mediators is often disrupted in solid tumors and is a hallmark of carcinogenesis [4].

Cancer cells blocked in the G1 cell cycle phase migrate and invade more than cells that undergo mitosis [30]. The presence of immune cells such as macrophages with a specific polarization phenotype can differentially modulate the growth and invasive phenotype of cancer cells. Our xenograft data suggest that presence of terminally differentiated macrophages lacking HO-1 suppresses tumor growth and induces E-cadherin expression. However, we did not find similar effect on EMT in TRAMP mice, where expression of E-cadherin correlated directly with Ki67 and tumor progression, and was higher in PIN lesions in TRAMP mice lacking HO-1 in myeloid cells. We speculate that a difference might be due to lack of T cells in the nude mice (xenografts) and therefore possible change in the infiltration of immune cells into cancer microenvironment. Further, the number of TAMs infiltrating xenografts as well as TRAMP tumors might be different. We injected terminally differentiated macrophages with cancer cells in xenograft model, in which we showed a direct influence of macrophages on cancer growth and EMT. In TRAMP mice, myeloid cells are incorporated during tumor growth and their phenotype and regulatory functions might be altered by cancer cells. These myeloid cells can be skewed to myeloid suppressor cells or TAMs, which support tumor growth. Characterization of the role of HO-1 in such conditions remains to be investigated in future study.

Increased expression of HO-1 restricts differentiation and polarization of TAMs which in turn regulate cancer cell invasion and growth. Myeloid HO-1 is known to drive macrophage differentiation [18]. Further, HO-1 knockout mice have decreased IRF3 function with defects in early immune responses [39]. We showed that HO-1 in macrophages is likely an important source of CO, which diffuses to the environment and targets E-cadherin in neighboring cancer cells. The role of HO-1 in modulating E-cadherin expression has been addressed in PC3 using heme treatment in a work by Gueron et al[1]. Overall heme treatment induced E-cadherin expression in cancer cells[1]. Overexpression of a truncated HO-1 (t-HO-1) lacking the TMS in HeLa and H1299 cells promoted cell proliferation and migration/invasion[2]. We showed that overexpression of either cytoplasmic or nuclear HO-1 in PC3 cells did not have significantly altered E-cadherin or Snail expressions (Figure 4D–4E). We speculate that a balance of cytoplasmic and nuclear HO-1 in cancer cells and myeloid cells derived HO-1 may dictate changes in E-cadherin expression.

E-cadherin plays a critical role in the function of the epithelial layer and is lost during EMT. E-cadherins are also recognized by Killer cell lectin-like receptor subfamily G member 1 (KLRG1) receptor on NK cells [42, 43]. NK cells in turn not only directly kill cancer cells or cells infected with virus but also generate cytokines such as interferon-γ which induces production of pro-inflammatory cytokines in the tumor stroma. INFγ is critical for M1 polarization of macrophages that inhibit tumor growth [44]. Alternatively activated M2 polarized TAM promote EMT in pancreatic cancers via activation of TLR-IL-10 pathway [45]. Lack of HO-1 correlates with less mature and polarized macrophages in tumor microenvironment, which decreases the number of NK cells being recruited to the tumor.

We observed a significant negative correlation of HO-1 in the cytoplasm and E-cadherin in cancer cells of specimens from prostate cancer patients. Presence of HO-1-positive macrophages in the tumor microenvironment might be an indicator of expression of E-cadherin and other EMT markers. HO-1 is a metabolic gene that controls energy balance via targeting of mitochondria through generation of CO [16, 38]. Assembly of E-cadherin based junctions requires energy and therefore the link between increased mitochondrial content in cancer cells and less aggressiveness of tumors may be important for therapeutic targeting. Depletion of ATP results in internalization of almost 70% of the E-cadherin from the cell surface [46] suggesting that energy from glucose metabolism is required for E-cadherin junctions. We showed that increased mitochondria activity in cancer cells via presence of macrophages leads to significantly increased E-cadherin expression. The presence of macrophages in co-culture with cancer cells leads to significant restoration of the spare respiratory capacity in cancer cells as compared to in culture of cancer cells alone. Dysfunction of the tricarboxylic acid (TCA) cycle induces tumor growth due to activation of a pseudo-hypoxic pathway under normoxic conditions [47, 48].

In summary, we showed that HO-1 in TAMs dictates cancer growth and metastases and as such can be a target for modulation of immune cell function during tumor dormancy and progression.

## MATERIALS AND METHODS

### Patient samples

36 human primary prostate cancers (PC) and 24 benign prostatic hyperplasia (BPH) samples were obtained from the cancer bank from the Department of Pathology at Lund University in Malmo, Sweden. Prostate cancer samples and BPH tissues from patients with Gleason grade 3–4 tumors and BPH samples were selected by certified pathologist (Dr. Martin Johansson, Lund University) for the tissue microarray (TMA) and printed in a TMA format in duplicates. Investigation has been conducted in accordance with the ethical standard and according to the Declaration of Helsinki and according to national and international guidelines and has been approved by the authors’ institutional review board.

### Geo data

GEO profiles from 18 normal prostatic tissues (without any pathological alterations), 62 tissues adjacent to tumors, 64 primary tumors and 24 metastatic samples were obtained from patients with prostate cancer as described in previously published data [26]. Specifically, 24 metastatic biopsies were derived from 4 patients with prostate cancer metastases to the liver, lymph nodes, kidney, lung and adrenal glands [26].

### Animal studies

Conditional HO-1 knockout *Hmox-1^fl/fl^* mice were crossed with *LyzM-Cre* mice to generate myeloid linage specific knockout of HO-1 (*LyzM-Cre:Hmox^fl/fl^*) as previously described [18, 40]. These mice were further crossed to TRAMP mice (*Hmox1^fl/fl^: LyzM-Cre:TRAMP*). Analyses of lesions in *Hmox1^fl/fl^: TRAMP* and *Hmox1^fl/fl^: LyzM-Cre:TRAMP* mice were performed at 25 weeks of age as previously described [15].

Xenografts (PC3 or A549) were established in Balb/c nude mice (Jackson Laboratory, Bar Harbor, Maine, USA). PC3 cells (2 × 10^6^) alone or PC3 with BMDM from *Hmox1^fl/fl^* (1:2 ratio) or PC3 with *LyzM-Cre:Hmox1^fl/fl^* (1:2 ratio) were injected to the right and left flanks of the nude mice to establish the tumors. A549 stably overexpressing HO-1 or control vector were injected to the flanks of nude mice. Syngeneic tumor model of Lewis lung carcinoma in *LyzM-Cre:Hmox^fl/fl^* or *Hmox^fl/fl^* mice were established by injecting 0.5 × 10^6^ cells into the flanks. Tumors, lymph nodes and livers/lungs were harvested for histology at 4 weeks.

### Cell culture, co-culture and treatments

Bone marrow derived macrophages (BMDM) were differentiated for 5 days in RPMI 1640 media with 15% fetal calf serum (FCS) and antibiotic-antimycotic solution supplemented with M-CSF (20 ng/ml). Polarization of BMDM was performed for additional 3 days RPMI 1640 media with 15% fetal calf serum (FCS) with 100 ng/ml LPS (Sigma–Aldrich, St. Louis, MO, USA) and 10 ng/ml IFNγ (PeproTech, Rocky Hill, NJ) for M1 macrophages and with IL-4 (10 ng/ml, PeproTech, Rocky Hill, NJ) for M2 macrophages. CO treatment was applied at 250 ppm in CO chamber (5% CO_2_, 21% O_2_ and 95% humidity) as previously described [18].

PC3 cells were co-cultured with BMDM from *Hmox1^fl/fl^ or LyzM-Cre:Hmox1^fl/fl^* mice in a 1:2 or 1:5 ratio for 24 h. PC3/BMDM cells alone or in co-culture were used for immunostaining using anti-E-cadherin antibody (CatNo: ab15148) and Mitotracker Red CMXRos (Life Technologies), for detecting mitochondria as well as for direct testing of mitochondrial activity Mitochondria Stress Kit (Seahorse) was used as previously described [49].

Overexpression of HO-1: Constructs were kindly provided by Dr. Chau (Taiwan) and forced overexpressions of Flag, HO-1-Flag or truncated HO-1-Flag (t-HO-1) were established in PC3 cells upon transfection with Amaxa. Forty eight hours after transfection, cells were stained or cell lysates were immunoblotted.

### Imunohistochemistry and immunostaining

Paraffin embedded formalin or zinc fixed tissues were processed for staining as previously described [15]. The following antibodies were applied: HO-1 (Abcam), MARCO (BD Biosciences), P-Stat3 (Cell Signaling), E-cadherin (Cell Signaling), F4.80 (BD Biosciences), Ki67 (Dako, Clone TEC3, rat monoclonal anti-mouse Ki67) and Gr1 (BD Biosciences). Briefly, slides were incubated with primary antibodies overnight at 4°C. After washing with PBS, slides with tissues were blocked with H_2_O_2_ followed by incubation with biotin-labeled secondary antibodies for 1 h at RT. VECTASTAIN Elite ABC System was used to enhance the signals (Vector Laboratories). DAB substrate (Vector Laboratories) was used for developing the reactions. After mounting the slides they were covered and analyzed by light microscopy.

Immunofluoresence staining was carried out as previously described [15]. Briefly, 2% paraformaldehyde fixation with subsequent washing, blocking and staining with primary and fluorescently labeled secondary antibody was applied (Alexa Fluor488 or Alexa Fluor594; Life Technologies, NY, USA). Nuclei were stained with Hoechst and immunofluorescence staining was analyzed by Zeiss Apotome fluorescence microscope.

### Immunoblotting

Proteins were harvested in RIPA lysis buffer (25 mM Tris-HCl, 150 mM NaCl, 1%NP-40, 1% sodium deoxycholate, 100 mM NaF, Complete Mini Protease Inhibitor Cocktail Tablets). After sonication lysates were centrifuged at 12000 × g, 4°C for 20 minutes. Protein concentrations were measured using BCA Protein Kit (Pierce). 15–35 μg were applied for NUPAGE SDS-PAGE Electrophoresis followed by transfer on the PVDF membrane (Amersham, US). Membranes were blocked in 5% non-fat milk for an hour. The following antibodies were used: P-Stat3 (Cell Signaling), E-cadherin (Cell Signaling), Twist-1 (Abcam), Snail (Santa Cruz Biotechnology), β-Actin (Sigma Aldrich), β-catenin (Cell Signaling). After washing with 1xTBS, membranes were incubated with HRP-conjugated secondary antibodies followed by detection of chemiluminescence on films (BioExpress).

### Statistical analysis

Statistical analysis was performed using SPSS 15.0 software (SPSS, Chicago, IL) or GraphPad (GraphPad Prism version 5.04, GraphPad Software, La Jolla California USA, http://www.graphpad.com). *T*-test (one- or two-tailed) and ANOVA were used to compare the groups and *p* < 0.05 was considered as significant. The correlation was calculated by Spearman's rank correlation and *p* < 0.01 was defined as significant difference between the studied variables.
